# Complementing Agents with Cognitive Services: A Case Study in Healthcare

**DOI:** 10.1007/s10916-020-01621-7

**Published:** 2020-09-15

**Authors:** Sara Montagna, Stefano Mariani, Emiliano Gamberini, Alessandro Ricci, Franco Zambonelli

**Affiliations:** 1 Università degli Studi di Bologna, Cesena, Italy; 2 Università degli Studi di Modena e Reggio Emilia, Reggio Emilia, Italy; 3 Maurizio Bufalini Hospital, Cesena, Italy

**Keywords:** Personal medical digital assistant, Trauma management, Cognitive services

## Abstract

Personal Agents (PAs) have longly been explored as assistants to support users in their daily activities. Surprisingly, few works refer to the adoption of PAs in the healthcare domain, where they can assist physicians’ activities reducing medical errors. Although literature proposes different approaches for modelling and engineering PAs, none of them discusses how they can be integrated with *cognitive services* in order to empower their reasoning capabilities. In this paper we present an integration model, specifically devised for healthcare applications, that enhances Belief-Desire-Intention agents reasoning with advanced cognitive capabilities. As a case study, we adopt this integrated model in the critical care path of trauma resuscitation, stepping forward to the vision of Smart Hospitals.

## Introduction

Several clinical problems require complex and quick decision making on diagnosis and care of patients. For instance, during trauma resuscitation, physicians need to decide and act in few instants. It is indeed demonstrated that trauma is a time-dependent pathology, where actions taken during the first hour, called “the golden hour”, heavily influence patient outcome [30]. Decisions are made by integrating information on patients’ biographical data, vital signs, lab tests, and imaging. However, these data are usually not available in the same location and at the same time, since interoperability between acquisition systems is still far from reality in many world countries—especially in primary care. Moreover, literature data and medical protocols must be taken into account to take the most informed choice: consulting them in these fast-paced scenarios, as well as remembering all of them, is simply impractical for a human being.

Pervasive computing can support physicians by acquiring patients’ data and integrating it with information accessed and analysed from literature, making them available anywhere, anytime. In particular, in this context, personal assistant agents (PAs) have been recognised as crucial components of a pervasive application [33]. Today, they commonly support users in daily activities, from simple ones such as make calls, read mails, send messages, open web pages, to more complex tasks such as schedule appointments, interact with physical objects in the environment, control smart devices [23, 26, 28].

In the context of healthcare, while intelligent agents have been applied for different purposes [15, 16, 22], the use of PAs has been poorly explored, mainly in the Ambient Assisted Living domain [32, 34] and in the remote management of chronic conditions, such as diabetes [4, 8]. However, the potential benefits of PAs to support physicians in their activities are unquestionable: the complexity given by the high variability and factors – of both users (patients) and environment – requires flexibility and situatedness, thus making modelling and engineering PAs very challenging. Identifying treatments that are personalised to each unique patient is the goal.

In [6, 7], the concept of *Personal Medical Digital Assistant Agent* (PMDA) has been introduced as a software agent supporting physicians’ activities. There, a PMDA was part of a pervasive computing application – the TraumaTracker system [27] – devoted at tracking medical activities during trauma resuscitation processes for real-time and accurate documentation. In [7], TraumaTracker has been extended adding a further level of assistance: the PMDA embeds and enacts knowledge (rules) for generating alerts on top of the collected information. It is based on the BDI (Belief-Desire-Intention) cognitive model and architecture [3, 11], that is: it acts according to predefined plans, encapsulating local rules and knowledge specified by the Trauma Team, with the purpose of generating alerts.

However, enhancing this local knowledge with a further level accounting for global knowledge – namely, for instance, best practices, clinical guidelines, population health, knowledge extracted from data on previous traumas – would empower PMDAs with the flexibility, dynamism, and awareness required to provide physicians with *real-time* effective assistance.

Recent advances of cognitive computing [18] make the notion of *cognitive services* or *cognition as a service* a reality [35]. They leverage complex algorithms from AI, enabling advanced analytical processing, sophisticated data discovery, and prediction generation. Some pilot studies in life sciences demonstrate that cognitive computing has the potential to extract information from the huge amount of data and literature available today, as never done before, thus expanding physicians knowledge base and expertise [2, 5].

In this paper we claim that integrating PAs and cognitive services can bring notable benefits and improve PMDAs reasoning capabilities in order to enrich the level of support in the healthcare domain. Accordingly, we propose an architecture where the PMDA interacts with a cognitive service that implements a Long-Short Term Memory network [12]: it receives as input time series data – with the main trauma resuscitation events and patient’s vital signs measures – and produces as output the prediction of risk for patient to go into shock. Such risk is integrated with the knowledge embedded in agent behaviour, and warnings are generated accordingly. LSTM is trained and validated with 573 reports acquired in 2018 with TraumaTracker in real trauma resuscitations. Dataset cardinality is under-sized with respect to the prediction problem the network is expected to solve. However, this paper is not meant to LSTM as shock predictors, rather to propose a new architecture for a PMDA dealing with trauma alerting.

The remainder of the manuscript is hence structured as follows: “Background” provides the necessary background on PAs, cognitive computing, and healthcare domain, “Principled integration” discusses integration opportunities in general terms, whereas “Proposed architecture in trauma resuscitation” describes the architecture adopted by ourselves to develop the system tested in the case study evaluated in “The case study of shock prediction”; finally, “Conclusion” provides final remarks and an outlook to further work.

## Background

### Personal assistant agents

Existing proposals and technologies have been developed for different kinds of purposes and capabilities, from scheduling joint activities (e.g., [40]), to monitoring and reminding users of key time-points (e.g., [38]), sharing information, assisting in negotiation and decision support (e.g., [21]).

Agents as personal medical assistant have been recently adopted also in the healthcare domain to support patients, physicians, or caregivers. The applications we found in literature are mainly devoted to support patients in their daily activities. They are usually part of Ambient Assisted Living applications [32, 34], where vital signs of patients and contextual information are acquired by sensors to provide PAs with all the data needed to devise out users needs and behaviour. Accordingly, they adapt themselves to improve given assistance, e.g. reminding users about medication schedule, identifying anomalies in patients’ health status, notifying caregivers when abnormal values are detected. For instance, [32] presents AMBRO, an IoT platform equipped with an “intelligent cloud system layer” where PAs learn on data and events acquired by the system and act by sending notification alerts to caretakers. Instead, [34] reports about the HERA (Home sERvices for specialised elderly Assisted living) project where a PA supports an elderly user – affected by chronic, Alzheimer, or mild cognitive impairment disease – following his/her daily routine and adapting its services to his/her habitual pattern.

### Cognitive computing

Proliferation of data collected with new mobile/wearable technologies opened a new era in computing where machine learning, natural language processing, and big data are integrated into the powerful framework of Cognitive Computing. Nowadays, IBM Watson is the major collector of cognitive computing technologies, which attracted widespread attention when in 2011 won against champions of US game show Jeopardy. In [17] author claims that Cognitive Computing success will be measured in terms of practical results, “like return on investment, new market opportunities, diseases cured and lives saved”.

The need and interest in exploring health applications is demonstrated by different cognitive healthcare solutions, such as the ones provided by IBM Watson Health. First studies discuss the potentiality of cognitive computing due to the enormous quantity of different data currently available and under-exploited—such as genomic data, exogenous data (acquired by Internet of Things technology) and electronic medical records [2, 5, 29].

### The healthcare scenario

The healthcare scenario is one of the most challenging contexts in which agent technologies may be applied: physicians have to deal with a multitude of situations, and the number of diseases and adverse physical conditions is so huge that is simply unfeasible for a human being to exhaustively recall everything anytime. More than that, each patient brings her/his complexity and singularities (demographic data, medical history, genetics and epigenetics, past life events, environment, mental status) that strongly impact their health. Physicians should consider all these variables to provide patients with efficient and successful diagnosis and care.

Given this enormous variability, medical errors are still dangerously common. They are due to human errors, but also to inefficiency of the care process and of hospitals’ organisation. According to [24], medical errors could be the third leading cause of death in the United States.

Preventing – or at least reducing – them is worthy and health IT (HIT) transformation has the potential to improve patient safety. We recognise two aspects of modern technologies that can strongly impact in this domain: *(i)* the computing power of modern mobile devices and wearable computing devices [25, 36, 37] (e.g., smart-glasses), their sensors equipment, and the possibility to interact with external services, are enabling factors to conceive a new generation of mobile/wearable PAs; *(ii)* big data computation and cognitive computing exploded in the last years, and advanced cognitive services are nowadays available [35]. Hence, caregivers can fruitfully collaborate with technology and get decision support in their activities without being obstructed.

## Principled integration

**Fig. 1:**
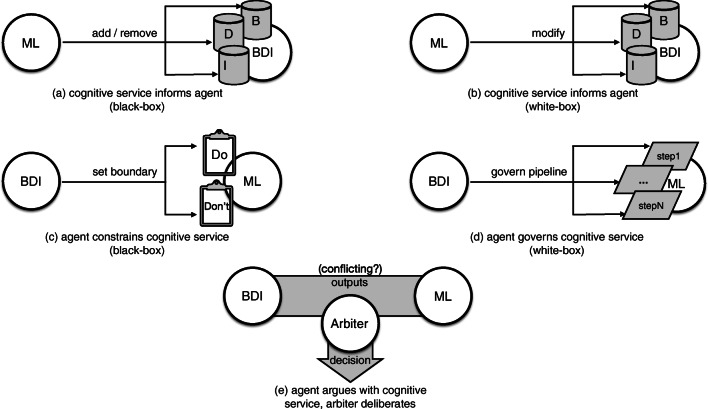
Possible integration architectures: **a** the cognitive service (ML) *manipulates* the agent (BDI) constructs by handling them as black-boxes (e.g. does not know how a plan is structured), **b** the cognitive service *modifies* the agent constructs internals (e.g. can change plans’ pre-conditions), **c** the agent *sets the boundaries* for the cognitive service’s operations, by filtering its outputs (e.g. ignores predictions with low confidence, filters out suggestions for unethical actions), **d** the agent *governs* the cognitive service’s operations, by executing its internal workflow (e.g. decides which prediction model to apply), **e** the agent and the cognitive service are peers engaging in an *argument* about what output to give (e.g. each supports its claims and attacks the other’s ones by providing suitable motivations)

A principled integration of cognitive services with an agent (in our specific case of machine learning with a BDI agent) requires first of all to enumerate the possible *architectures* supporting such integration, in terms of roles, responsibilities, and goals of collaboration ascribed to each participating component. Figure 1 depicts such an enumeration. We emphasise this is not about the software architecture actually implementing *technical* integration between the different software pieces involved; rather, it is about the ways in which the agent abstraction and the concept of cognitive service (as derived from other research areas of AI, such as machine learning) can coherently co-exist and co-operate to deliver decision support—a specific case study in healthcare, relying on architecture (a), is described in “The case study of shock prediction”.

The first possible architecture, depicted by case (a) in Fig. 1, assumes the cognitive service (ML, as for Machine Learning component) to act as a *supervisor* overseeing agents’ operations, and influencing the latter activities by manipulating its fundamental constructs, such as adding goals, removing behaviours, or refining knowledge. Notice that the agent may be unaware of the cognitive service supervision. The goal of such a form of collaboration is that of enabling *adaptiveness* of the agent, by letting a cognitive service expand or shrink the range of possible activities undertaken by an agent, for instance by adding goals, plans, or beliefs synthesised from collected data about either the domain of operation (e.g. temporal evolution of a patient’s clinical conditions) or the monitoring of agent action (e.g. failed plans, ineffective actions, etc.). A fundamental characteristic of such an architecture is that the cognitive service sees the agent’s fundamental constructs as *black-boxes*, that is, it cannot modify their internals—for instance, in the case of integration with a BDI agent, the cognitive service can add plans but cannot modify plans’ pre-conditions.

The second possible architecture, depicted by case (b) in Fig. 1, is similar: it differs solely for the fact that in this case the cognitive service has full awareness of the agent’s constructs internals, hence can operate on them, too—in other words, they are *white-boxes* to the eyes of the cognitive service. In the case of a ML component overseeing a BDI agent, this means that the former can, for instance, adapt the pre-conditions of a plan during time to better reflect an ever-changing situation (e.g. worsening of patient’s conditions). The roles (supervisor/supervised) and collaboration goals (adaptiveness) remain unchanged with respect to case (a). Even if such a difference may appear negligible, it is not: it necessarily requires a tighter integration between the agent and the cognitive service, as awareness of the former internals by the latter must be greater than in the previous case.

It is worth emphasising that the aforementioned alternatives both ascribe the responsibility of supervision and intervention to the cognitive service, whereas the agent is passively (possibly, unawarely) supervised, hence influenced. In cases (c) and (d) depicted in Fig. 1 such roles are inverted.

In fact, the third possible architecture depicted by case (c) in Fig. 1 ascribes the supervisor role to the agent, which is in charge of drawing the *boundaries* within which cognitive service’s outputs are admissible. In other words, the agent is given the responsibility to guarantee that the outputs of the cognitive service are actually considered by the application at hand only and only if they conform to a series of “safety checks” (e.g. drug administration does not violate safety thresholds established by known guidelines)—actually operating as a filter. Again, it is worth noting that the cognitive service may be unaware of being supervised. The goal of such a form of collaboration is that of ensuring *safety* of the overall decision support system (i.e. the cognitive service and the agent considered altogether), by letting the agent enforce rules embedded in its knowledge base (e.g. in the form of beliefs in case of a BDI agent) expressing the “do and don’t” to guarantee for the application domain—e.g. *never ever* administer drug *D*_1_ together with drug *D*_2_, or *always* schedule intervention *I*_2_ if intervention *I*_1_ has been performed. Finally, as for case (a), a peculiar trait of this integration architecture is that the agent has no knowledge of the internals of the cognitive service, which appears to it as a black-box.

Case (d) may appear similar to case (c), but is in fact quite different: not only here the agent handles the cognitive service as a white-box, by being able to operate on its inner workflow (the ML “pipeline” as it is commonly called), but also the goal of collaboration may now encompass adaptiveness, too—in addition to safety. What remains identical, instead, is the supervision role played by the agent with respect to the cognitive service: the former oversees the latter operations and intervenes by changing working parameters or by directing workflow steps. For instance, in the case of a ML pipeline including hyper-parameters search and models selection, the agent may apply different scoring metrics and search coefficient to then select the best performing one. Once again, this integration architecture requires tighter coupling between the cognitive service and the agent, as the latter needs greater awareness of the former internals with respect to previous case.

It is worth emphasising that in each of the four proposed architectures the participants to the interaction are not peers on an equal level, but a subordinate relation is always present: for cases (a) and (b) of the agent with respect to the cognitive service, the contrary for cases (c) and (d). The last integration architecture si different in this aspect.

In case (e) of Fig. 1, in fact, the agent and the cognitive service are *peers* of a collaborative architecture where they both provide their outputs to an *arbiter* responsible for making an ultimate decision about the insight to give outside the system. The goal of this architecture is to enable adaptiveness, safety, and any other property desirable for the application at hand through the use of *argumentation*: when both the agent and the cognitive service have an insight to give, they engage in an argumentation dialogue governed by the arbiter, who may request additional explanations about the given insight to both parties. For doing so, any known argumentation framework for negotiation could be used [31]. It is also worth emphasising that this solution is loosely inspired to the “arguing machines” model described in [9].

We want to stress that each of the alternative architectures pose several *technical challenges* to software designers, which we do not discuss here for the lack of space, except for our choice regarding the case study presented in the following sections. For instance, whenever agency is involved, considering the impact of integration with other paradigms / technologies on agents’ *autonomy* should always be a primary concern. Also, we do not claim that the enumerated architectures are new; on the contrary, some of them may actually embed the principles followed by some existing research works: for instance, literature on belief revision [14, 20] or on automated planning [19, 41] may be framed in our cases (a) and (b) depending on how tight coupling is. Other research themes such as reinforcement learning and the emerging XAI (eXplainable AI) paradigm may find a fit in Fig. 1 as well.

Finally, we think it is worth to emphasise that choosing the best architecture depends on many heterogeneous factors, there including the degree of explainability desired, which is typically high in the healthcare domain. Although providing guidelines for choosing the best architecture obviously depend on how “best” is defined, which is out of the scope of this paper, and would likely require its own manuscript, we feel to assess what follows: it is likely that integration architecture (e) will provide the greatest explainability. In fact, the argumentation-based setting itself guarantees that only insights with sufficient arguments are delivered outside the system. As far as the other architectures are concerned, cases (c) and (d) also guarantee some level of transparency, as the goal-oriented and beliefs-driven nature agency is well-suited to lend itself to human interpretation, whereas cases (a) and (b) inevitably depend on the specific ML models adopted, as some are explainable (e.g. decision trees) and some others are not (e.g. neural networks).

## Proposed architecture in trauma resuscitation

In the following, we present a model for the integration between BDI agents and ML algorithms relying on a specific case study devised to manage issues related to the trauma resuscitation activities within an Emergency Department.

### Existing system

In [6] and [7], a *Personal Medical Digital Assistant* called Trauma Assistant Agent has been presented, as an agent with the goal of autonomous documentation and reporting, and automatic alerts generation. Using BDI model of agency, rules were naturally and effectively formalised in terms of agent plans, exploiting agent’s beliefs to generate alerts. Table 1 shows, as an example, two of the rules authors presented in [7], specifying when the alert is generated, what alert message is displayed, and the rationale for the rule (described in the caption).

**Table 1:** An extract of rules used by the TraumaTracker system in [7] for generating alerts to be notified to the Trauma Leader and its team. Rule #1: The Early Coagulation Support prescribes the administration of both fibrinogen and tranexamic acid in the case of blood transfusion during a trauma; Rule #6: In the case of fracture exposition Antibiotic Prophylaxis should be performed as soon as possible

Rule	Condition	Alert message
1	Zero Negative Blood administered at time *t* *but* at time *t* + 5*min* not administered fibrinogen and tranexamic acid.	“Administer Fibrinogen and Tranexamic Acid” for 10 secs
6	If there is a fracture, when exiting from the Shock Room without having started the antibiotic prophylaxis	Message: “Activate Antibiotic Prophylaxis?”

As a simple example, rule #6 is implemented by means of the following plan (in pseudo-code):

+ev(room_out(shock_room),T) : fracture(true) ∧ $\nexists $ev(drug(abt),_) → +alert(checkABT,T).

That is: when the agent has a new belief about the patient exiting the shock room with a fracture diagnosis, and no belief about administration of Antibiotic Prophylaxis, then a new belief about an alert to be generated is produced, as a reminder to activate Antibiotic Prophylaxis.

However, the agent does not plan from first principles. Instead, it is equipped with a library of pre-compiled plans. These plans are manually constructed, in advance, by the agent programmer: alert generation is driven by a set of rules defined a-priori by the Trauma Team. Rules have been designed after a retrospective analysis of reports, in order to improve the performance/quality of their actions, preventing dangerous situations for the patient.

### New system

The goal is to design a flexible PMDA that, interacting with a cognitive service, adapts alerts to the specific patient history and context. Accordingly, the set of agent’s beliefs, and possibly plans, is updated real-time *during* the process of trauma resuscitation.

**Fig. 2:**
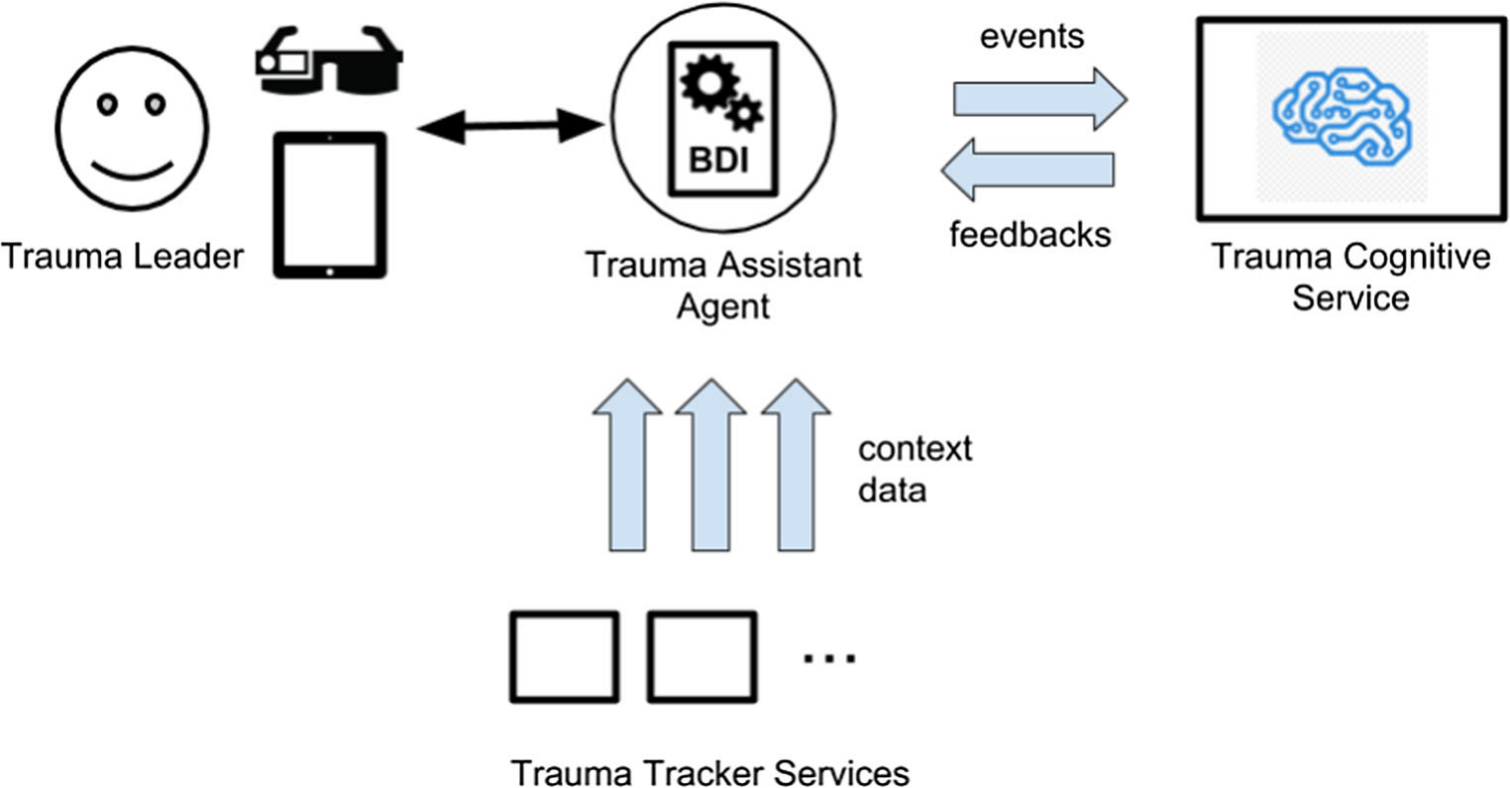
Integration model

The integrated model is an example of the first possible architecture, depicted by case (a) in Fig. 1. It is based on the continuous interaction between the Trauma Assistant Agent and a service – referenced in the following as Trauma Cognitive Service – installed and running in the Hospital intranet, among existing services available to support Trauma tracking and assistance (see Fig. 2). We chose architecture (a) as we wanted to complement the set of rigid and pre-determined alerting rules embedded in the agent plans library according to expert knowledge, with a more dynamic set of rules depending on the possible unpredictable situations which could arise during an ongoing trauma, whose detection is duty of the Trauma Cognitive Service. We didn’t chose architecture (b) as we wanted to keep clearly separated the rules stemming from expert knowledge from those automatically crafted by the Trauma Cognitive Service, so as to guarantee to the medical staff maximum transparency, hence accountability, of what is due to black-box AI, and what instead stems from human knowledge. We didn’t chose architecture (e) as in our target use case there is no automatic intervention, only suggestions (in the form of alerts), hence there is always a human being (the medical staff) acting as arbiter to choose the most appropriate solution (for instance, whether to actually consider the alert, or not)

During the management of a trauma, Trauma Assistant Agent collects and tracks data related to all relevant events, including the Trauma Leader inputs (by means of the tablet) and information retrieved from services on the Intranet that are part of the TraumaTracker ecosystem. Information automatically retrieved are:

- the Location Service provides information about the current location (room) of the patient—by exploiting a beacon-based infrastructure;
- the Vital Signs Monitoring Service provides information about the current vital signs of patient—fetched from the existing Hospital Vital Sign infrastructure technology.^1^

All these data – those entered by the Trauma Leader and those retrieved automatically – are collected in the belief base of the agent, with a twofold purpose: *(i)* they are used to support local reasoning of our PMDA, the Trauma Assistant Agent, and the generation of alerts by means of the specific plans, and *(ii)* they are sent to a service keeping track of the information about an ongoing trauma, referred as Ongoing Trauma service.

Then, the information about an ongoing trauma are asynchronously accessed and monitored by the Trauma Cognitive Service. Such a service implements a set of algorithms from Machine Learning and Deep learning to analyse and reason on the incoming data, that represent the current situation of the patient, but also the temporal evolution of patient’s vital signs and the track record of the actions taken by physicians since the beginning of the trauma resuscitation. These temporal data characterise well the clinical condition of the patient and, if properly elaborated, may be used to make predictions on the forthcoming clinical state, to identify the most common actions taken in similar previous traumas, as well as correspondent outcomes, and to identify guidelines inspecting literature knowledge and clinical protocols.

The Trauma Cognitive Service has been trained with data archived in the Trauma Store service, which is the Knowledge Base representing the shared data base of all trauma. Reasoning eventually provides a set of new feedbacks, e.g. further alerts or clinical indications, that are sent to the Trauma Assistant Agent. The Trauma Assistant Agent then integrates the alerts locally generated, according to its own plans, with those generated by the Trauma Cognitive Service. Accordingly, the proposed integration follows architecture (a) in Fig. 1, as the cognitive service adds beliefs and plans to the agent, even though no supervision is enacted.

## The case study of shock prediction

We evaluated the model presented in “Proposed architecture in trauma resuscitation” for alerting physicians on the risk of shock. Shock is a critical condition potentially life-threatening. According to [10], it is defined as:

… state of cellular and tissue hypoxia due to reduced oxygen delivery and/or increased oxygen consumption or inadequate oxygen utilization. This most commonly occurs when there is circulatory failure manifested as hypotension (ie, reduced tissue perfusion).

**Fig. 3:**
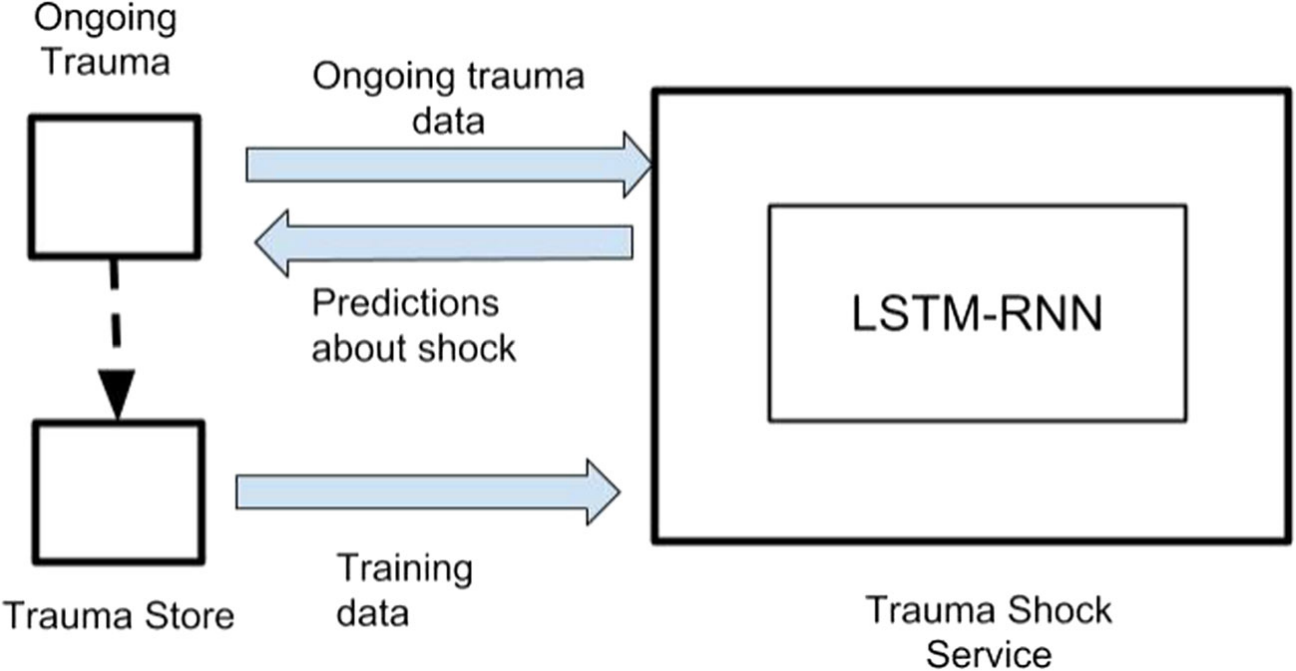
Shock Service interacting with Trauma Assistant Agent

Knowing in advance the risk of shock is crucial to decide the next actions to take such as, for instance, if patient can be taken to imaging for Computed Tomography (CT). Accordingly, we present a specific kind of Trauma Cognitive Service, the TShockService (see Fig. 3).

PMDA receives as input a report with patient’s information, trauma dynamic, and previous events occurring during trauma resuscitation which has the form—extracted from a simulated report:

2018-11-12Patient DataGender MType AdultAge51Trauma InformationInjurity Severity Score (ISS) Total ISS: 18ReportShock-Room06:05 Patient enters in Shock-Room06:15 Drug: Crystalloid 500ml06:18 Drug: Fentanil 100mcg06:18 Diagnistic Exams: Echofast06:18 Diagnistic Exams: Chest x-Ray06:18 Diagnistic Exams: ABGCT06:45 Diagnistic Exams: cerebral-cervical CT

Reports abound in useful information and fill a rich database of traumas. Generally speaking, such a data source can be useful for predicting complications, and for suggesting physicians on the next action to do according to what has been done in similar conditions.

The goal is to find which sequences of events are informative for predicting shock. Since we are interested in analysing time series data, we adopt *Long short-term memory* (LSTM) [12] that are known to be the most effective sequence models among the family of Recurrent Neural Networks (RNNs) [13]. A key feature of this family of neural networks is that they include feedback loops to integrate information from previous steps with most recent data. In [1] a RNN has been designed to process the course of patients in a Pediatric Intensive Care Unit with the goal of predicting patient mortality. In [39] LSTM are used to early detect bloodstream infections in Intensive Care Unit (ICU) from time series of 9 clinical parameters selected as features.

### LSTM settings

We make our PMDA interacting with a service where LSTM algorithm has already been implemented.

We used a 3 layers LSTM: an input layer with 60 neurons, a hidden layer with 40 neurons – representing 2/3 of input neurons as for standard configurations – and an output layer with 2 neurons.

**Fig. 4:**
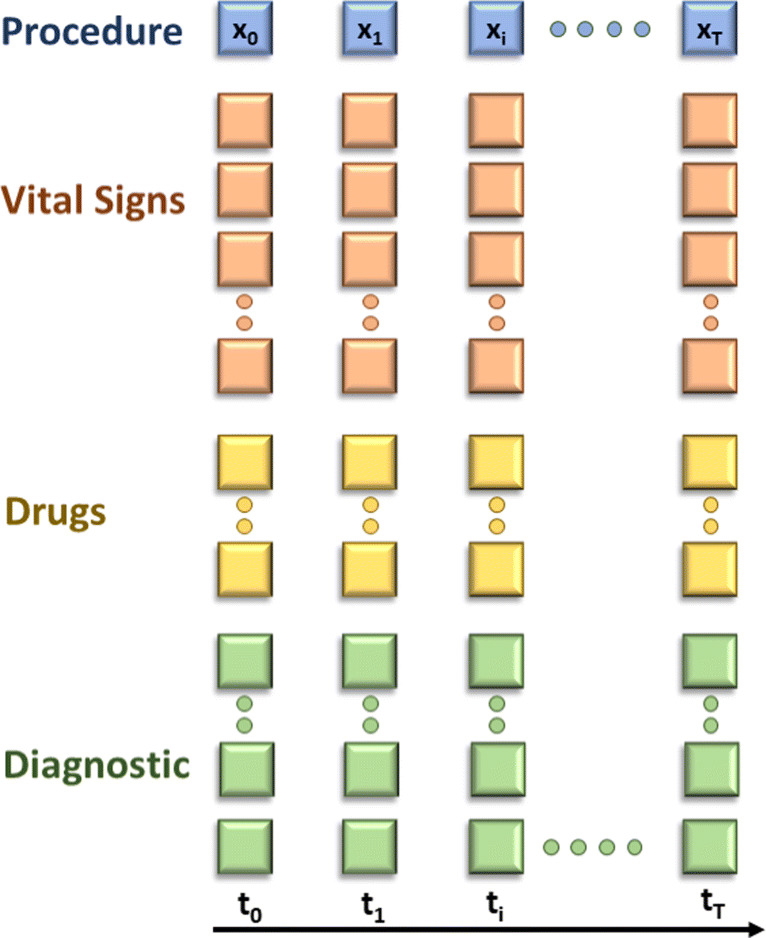
LSTM input matrix: each column represents network inputs at each time step, while the *x*-axis displays the time steps

Network’s input is a matrix **X** containing diverse data related to clinical measures on patient and to the trauma resuscitation process (rows) at different timestamp (column). As depicted in Fig. 4, each column *x*_*i*_ contains the following features, that can be semantically grouped in 4 different categories:

Vital Signs: — A set of 38 features measuring vital parameters (*e.g.*, blood pressure, SpO2, EtCO2, hearth rate, temperature), neurological examinations (*e.g.*, GCS, eyes deviation, pupils), airways. They are repeated at every timestamp *t*_*i*_ and refer to the most recent measurement. They change once new values are available.
Diagnostic exams: — A set of laboratory results: 7 features from Arterial-Blood Gas (ABG) test (*e.g.*, pH, Lactates, Base Excess, glycemia) and 3 from rotational thromboelastometry (ROTEM) (fibtem, extem and hyperfibrinolysis). We did not include imaging since, according to clinicians, it does not provide information useful for shock prediction. As for vital signs, they have the last value measured at each timestamp.
Procedures: — 1 feature is dedicated to possibly indicate the id of the specific medical intervention patient received at time stamp *t*_*i*_, such as intubation, fibroscopy, drainage.
Drugs: — 11 features in the vector are drugs. We have “one-shot” or “continuous-infusion” drugs. If one-shot, 2 components are filled, one for the drug id, the other for the dose. For continuous infusion we have the dose per time unit. If none, the field has default value 0.

Network output represents the two classes shocked/not-shocked. Since LSTM allows the user to specify how long into the future predictions are for, our LSTM predicts the probability that a patient will go into shock in the next 15 minutes. We choose this time-frame since it is the average duration of imaging examinations, such as CT, where it would be difficult for physicians to promptly operate.

### LSTM training

We leveraged 573 reports acquired with TraumaTracker system. Data have been automatically labeled in the two classes: patients with a systolic blood pressure under 90 or treated with crystalloid, adrenaline-continuous-infusion, noradrenaline-continuous-infusion, dopamine-continuous-infusion, blood cells, colloids are labeled as shocked patients. Among the 573 patients, 329 went into shock during trauma resuscitation. The input matrix has the form [$x_{0},\ldots ,,x_{i},x_{t\rightarrow shock}$] for shocked patients, and the form [*x*_0_,…,*x*_*i*_,*x*_*T*_], where *x*_*i*_ is the feature vector at timestamp *i* and 0-*T* are meant as the starting and ending time of the resuscitation process. 90% of these reports has been used during the training phase, where *T* ∈ [1, 37] and a mean number of events equal to 4.18.

### LSTM validation

Remaining 57 reports have been used in the validation phase. They account for 578 events. Network prediction accuracy has been computed as 0.8391. In particular, for the Shocked class, we have precision 0.6787, recall 0.6141, and F1 0.3586. In Table 2, we reported the confusion matrix obtained during the validation phase. It reports TP, TN, FP and FN once trying to predict next event of each of the 578 ones. Although the overall accuracy is not bad, from these preliminary results we can observe that LSTM mainly fails in predicting shock events. Recall and F1 can be improved once more data will be collected.

**Table 2:** Confusion matrix

	No-shocked	Shocked
No-shocked	459	28
Shocked	65	26

Moreover, even if we expect to improve these results with a bigger dataset, our goal here is not to show a case study where LSTMs prove to be the best choice – among all the deep learning algorithms –, rather to demonstrate the feasibility of the integrated architecture and the different and complementary roles that the two components, relying respectively on knowledge-based plans and on data elaboration, have.

## Conclusion

In this paper we presented a first example of integration of PMDA and cognitive services, specifically devised for healthcare applications. To discuss the proposed architecture we made use of a real scenario in trauma resuscitation: a PMDA interacts with a cognitive service that predicts the risk of shock in the next 15 minutes, providing alerts to physicians in case of high risk.

This is a first step that paves the way for a promising research direction towards devising a novel BDI architectures where agent’s reasoning is empowered with cognitive computing. Such a model is crucial for designing effective PMDA since supporting caregivers with a real time, comprehensive and flexible assistance can contribute in reducing those medical errors that are still dangerously common.

## Footnotes

1 Based on Draeger Infinite Gateway.
